# Case of Aneurism by Anastomosis, in Which Both the Primitive Carotid Arteries Were Tied

**Published:** 1830-06

**Authors:** R. D. Mussey

**Affiliations:** Professor of Anatomy and Surgery in Dartmouth College, Hanover, New Hampshire.


### ANEURISM BY ANASTOMOSIS.

*Case of Aneurism by Anastomosis, in which both the primitive Carotid Arteries were tied.* By R. D. Mussey, M.D. Professor of Anatomy and Surgery in Dartmouth College, Hanover, New Hampshire.*

J. Pattee, aged twenty years, consulted me in September, 1827, respecting a pulsating purple tumor, situated upon the vertex of his head, with a base of about five inches in diameter, and rising an inch and a half or two inches above the cranium. This tumor had existed from infancy, but had greatly increased within the last two years. Upon the apex of the tumor was a sluggish ulcer, of an inch in diameter, which commenced two years before, had been slowly enlarging, and which had bled occasionally during the preceding spring and summer, and once to the amount of two quarts, as estimated by his physician.

The left temporal artery and vein, where they pass in front of the ear, presented through the integuments the appearance of a vessel five eighths of an inch in diameter. This was so prominent in its winding course along the temple, and even to the base of the tumor, that its pulsations could be distinctly seen at the distance of *fifteen* feet. A vein which passed from the tumor down to the forehead, was full and prominent, and half an inch in diameter; and when the head was shaved, more than *twenty* arteries running to the tumor were seen actively pulsating, none of which, as they appeared through the integuments, were less than a middling-sized goose-quill.

Curious to know what would be the effect of securing the large arteries, from which branches were distributed to the tumor, I tied, on the 20th of September, the left primitive carotid. The tumor, after the operation, was a little less tense, and less livid; still the active pulsation of the numerous arteries upon the right side of the base of the tumor rendered it evident that there was an adequate supply of blood. On the twelfth day from the operation, I tied the right primitive carotid artery. The face was a good deal paler immediately after this operation, but, what was scarcely to have been expected, the functions of the brain were not apparently disturbed. There was neither nausea nor faintness; the patient rose from the table, stood up, and while standing put on his vest and coat, and tied on his cravat; he then walked down two flights of stairs, got into a

* American Journal of Med. Sciences.

carriage, and rode to a distant part of the village, without feeling the least symptoms of faintness, or manifesting signs of inconvenience.

The tumor, which, after this operation, was daily dressed with a compress and bandage, so as to make slight compression upon it, the compress being kept constantly moist with alum water, progressively subsided, and in about four weeks was reduced apparently to about one third of its original volume. At this period the tumor became stationary, and in five or six days began very slightly to enlarge; its colour was a little deepened, and a feeble thrill, corresponding with the pulse in other parts, could occasionally be perceived in the left temporal artery. These appearances indicating that nothing further was to be expected from tying the carotid, or from astringent applications or compression, I proceeded, on the 22d of November, about six weeks from the second operation, to remove the tumor.

This was accomplished by first encircling the tumor by an incision made quite through the soft parts, and rapidly dissecting away the whole mass from the pericranium. More than an hour was occupied in carrying the knife around the base of the tumor; the whole operation being conducted with immediate reference to the saving of blood. Not more than an inch and a half of the scalp was divided at a time, and immediately upon the division, firm compression was made upon each lip of the incision, while the vessels were secured by ligatures, more than forty of which were applied in going round the tumor. Notwithstanding, however, these precautions, it was estimated by all present that blood to the amount nearly of two quarts was lost during the operation. The patient was faint, and continued very feeble for several hours. The naked pericranium, in extent equal to about twenty-five square inches, granulated kindly, and in eight weeks the wound was nearly healed. It was some months, however, before the cuticle, through its whole extent, became firm, so as to sustain itself under considerable variations in the state of the circulation. The patient returned to active labour upon a farm the following March or April, has continued it ever since, and has been one of the most athletic and industrious labourers I have seen.

This case is interesting in a physiological view, for at no period subsequently to the operation of tying the second carotid, with the exception of the faintness and debility which occurred from the actual loss of blood on the removal of the tumor, has there been a single symptom of deficiency of blood in the brain. Indeed, at one period, viz. in the spring of 1829, sixteen or seventeen months after the operation, the opposite state seems to have existed; as the patient had flushed face, accompanied with the headach daily for two or three weeks, and was not relieved essentially by cathartics. A single large bleeding entirely removed the symptoms.

